# Trust and respect in the patient-clinician relationship: preliminary development of a new scale

**DOI:** 10.1186/s40359-019-0347-3

**Published:** 2019-12-30

**Authors:** Paul Crits-Christoph, Agnes Rieger, Averi Gaines, Mary Beth Connolly Gibbons

**Affiliations:** University of Pennsylvania, 3535 Market St (Room 650), Philadelphia, PA 19104 USA

**Keywords:** Trust, Respect, Alliance, Psychotherapy, Scale development

## Abstract

**Background:**

Trust and respect may be an important component of client-provider relationships. This study aimed to develop and report preliminary psychometric analyses of a new brief measure to evaluate a patient’s level of trust and respect for their clinician. The scale was designed to be applicable in multiple healthcare contexts, with a particular focus on mental healthcare.

**Methods:**

Adult patients completed the study survey in an academic outpatient psychiatric clinic waiting room. Classical and Item Response Theory (IRT) analyses were utilized to examine the adequacy of scale items. Validity was examined in relation to the patient-therapist alliance and to willingness to share private information (social media content) with one’s clinician.

**Results:**

Beginning with 10 items, a final 8-item version of the measure was created with an internal consistency reliability of .91. Principal components analysis indicated that the scale was best viewed as capturing one overall dimension. A Graded Response Model IRT model indicated that all items contributed information on the latent dimension, and all item curves were not flat at any region. The correlation of the trust/respect total score with the alliance was .53 when respect-related items were deleted from the alliance score. The trust/respect scale was significantly associated with patient willingness to share social media posts with their clinician but the alliance was not.

**Conclusions:**

The brief measure of patient trust and respect towards their clinician was unidimensional, showed good internal consistency, and was not redundant with existing measures of the alliance. The scale has the potential to be used in a wide variety of healthcare settings.

## Background

Trust has been described as perhaps the most important ingredient for the development and maintenance of happy, well-functioning relationships [1]. Indeed, major theories of human psychosocial development such as Bowlby’s [2] attachment theory and Erikson’s [3] theory of stages of development emphasize the idea that trusting relationships early in life build a foundation for better functioning in adulthood. Despite the prominence of Bowlby’s and Erikson’s theories, and the importance of trust within such theories, there has been surprisingly little empirical research on trust in close relationships. The existing empirical studies on trust in the personality and social psychology literature have provided evidence that degree of trust in close relationships maps onto attachment patterns. For individuals with a secure attachment style, compared to those with an insecure attachment style, greater trust is associated with more constructive coping strategies where there is a violation of trust in love relationships [4]. In addition, within close relationships, individuals develop trust in their partners when they perceive that their partners have demonstrated pro-relationship behaviors rather than only behaviors that show self-interest [5].

Such findings from personality and social psychology have clear potential relevance to patient-provider relationships, particularly the relationship between patients and psychotherapists. For example, level of patient trust in the therapist may be critical to working through ruptures in the therapeutic relationship. Extrapolating from the findings of Wieselquist et al. [5], it seems likely that therapist actions that are perceived by the patient as pro-relationship such as self-disclosure, fee reductions, and referrals for additional services, might be particularly useful for building trust in psychotherapy. However, there has been little research specifically on the patient level of trust toward psychotherapists.

Relevant to the concept of trust is the literature on the therapeutic alliance in therapy. In his seminal paper defining the alliance, Bordin [6] discusses the bond component of the alliance in the following way: “Some basic level of trust surely marks all varieties of therapeutic relationships, but when attention is directed toward the more protected recesses of inner experience, deeper bonds of trust and attachment are required and developed” (p. 254). Consistent with Bordin’s [6] discussion of the bond, virtually all alliance scales contain at least one item referring to mutual trust. “Mutual trust” is likely overlapping, but not identical to, the patient’s individual level of trust towards the therapist. Moreover, such scales also include, and are actually primarily composed of, items not specifically related to trust. For example, the following items are included within the Bond subscale of the Working Alliance Inventory (WAI) [7] client long-form version, in addition to mutual trust: comfort with therapist, mutual understanding, liking of therapist, genuine concern by therapist, therapist appreciation, importance of relationship, feeling cared about, and feeling that if one says or does the wrong things the therapist would stop working with the patient. Thus, while the WAI Bond scale includes an item on mutual trust, the scale is not geared toward more specifically investigating patient level of trust in their therapist.

Still, given the inclusion of trust items within alliance scales, the empirical alliance literature has some bearing on the role of trust in psychotherapy. Although some research has suggested that, at least within the context of cognitive therapy, agreement on tasks and goals is the critical aspect of the alliance that leads to positive outcomes [8], other research has emphasized the importance of the bond component as a predictor of psychotherapy outcome [9].

Such findings regarding the bond component of the alliance are consistent with Bordin’s [6] theorizing on the importance of mutual trust in therapeutic relationships. However, it is not clear from the existing literature which facet of that bond is critical to positive treatment outcomes. Many studies only focus on the total alliance as an amalgamation of many relationship qualities, rather than on specific subcomponents of that relationship. As described above, the Bond scale of the WAI includes a diverse (though correlated) set of items, and its subscales are composed of a mixture of items that have a focus on mutual feelings (e.g., mutual trust) and items that highlight individual feelings (e.g., patient’s view of the therapist’s honesty). Thus, the extent to which the patient’s feelings/beliefs towards the therapist, versus a sense of mutual feelings/beliefs, is critical in the process and outcome of therapy is not clear when using alliance scales, including the WAI.

Beyond the alliance, a few studies have focused more specifically on trust in the patient-therapist relationship. One study of 17 clinicians and 48 patients by Peschken and Johnson [10] used a modified version of the Dyadic Trust Scale [11] that was developed for measuring trust among intimate partners. The study found that therapist ratings of trust in their clients correlated positively with therapist ratings of facilitative conditions, and that client ratings of trust in their therapists correlated positively with client ratings of facilitative conditions. But, therapist ratings of trust did not correlate with client ratings of trust, and neither set of ratings correlated significantly with current symptom levels. Limitations of this study include the fact that clients and therapists rated trust in general, not current (i.e., session or weekly) level of trust, the use of a modified scale designed for use with intimate partners without validation for use in therapy, and the lack of examination of trust in relation to outcome of treatment. The lack of a significant relationship between therapist trust of their client and client trust of their therapist is of particular interest, as it suggests that these two constructs should be measured separately. Along these lines, an early study found that therapist self-disclosure, which one can hypothesize is likely more common when the therapist trusts the client, actually has a negative association with client trust of the therapist in an analogue therapy interaction [12].

Another measure of client trust of their therapist is the “trustworthiness” subscale included within the Counselor Rating Form (CRF) [13]. The CRF has been used by observers rating tapes of psychotherapy (e.g., [14]) and by patients in treatment rating their counselor (e.g., [15]). Two studies, however, have concluded that a separate trustworthiness factor is not apparent in the CRF [16, 17], further suggesting that existing measures are not sufficient in capturing the construct of patient trust in their therapist. In addition, the CRF has been found to correlate very high (0.78) with the alliance [18]. Though some degree of overlap with the alliance is to be expected, ideally a scale measuring patient trust and respect towards their clinician would not be redundant with alliance scales that measure agreement on tasks, agreement on goals, and strength of the bond as defined as mutual trust, respect, and liking.

Separate from research on psychotherapy, there has recently been considerable attention paid to trust in the patient-clinician relationship within the context of primary care and other medical interactions [19, 20]. Trust in physicians in the United States is notably a concern. A survey found that the United States was tied for 24th place internationally in terms of the proportion of adults who agree with the statement, “All things considered, doctors in [your country] can be trusted” [21], with only 58% of adults in the U.S. agreeing with the statement. Over the years, a variety of scales have been developed for measuring trust in the context of medical care interactions (e.g., [22–24]). The items of these scales, however, often focus on characteristics of medical doctors that might further trust (e.g., “sometimes you worry that your doctor’s medical decisions are wrong,” “your doctor has better medical skills than most other doctors in his or her field”). Such scales may be measuring aspects of provider competence that may, or may not, further trust in the relationship, rather than directly measuring the interpersonal and emotional dimension of trust. One can imagine a provider who competently accomplishes needed tasks for a patient, but for whom the patient still does not have a strong feeling of trust towards. Moreover, the types of behaviors that primary care doctors perform (reflected in items such as “your doctor would never prescribe the wrong medicine for you”) have less applicability to psychotherapy, especially when conducted by non-M.D. providers.

A related construct to trust is respect. In general healthcare settings, the patient’s perception of whether their doctor displays respect towards them has been found to be the best predictor of patient’s overall rating of their view of their doctor [25]. In psychotherapy, the importance of a therapist adopting a respectful view of their patients can be traced back to Carl Roger’s [26] central concept of unconditional positive regard. Surprisingly, few quantitative studies of psychotherapy have explicitly focused on the patient’s reported respect for their therapist. However, a review of 13 qualitative studies found that disrespectful behaviors by therapists were one important barrier to the formation of a positive therapeutic relationship [27]. Thus, measuring patient respect for their clinician might provide a window into the development of a positive relationship and consequently better outcomes.

Most alliance scales contain one or two that specifically focus on respect. The WAI has an item on mutual respect that is included within the Bond scale. Given the definition of respect as “a feeling of deep admiration for someone or something elicited by their abilities, qualities, or achievements” [28], one can imagine a patient who respects a clinician (e.g., because the clinician has received training from the best Universities, written books, received accolades, etc.), but has no idea as to whether that clinician in turn respects them and therefore mutual respect is not rated highly on the WAI. Furthermore, it might well be the case that some patients have some level of respect for their clinician based on professional credentials and accomplishments alone, but a high level patient personal respect for that clinician may or may not be present. Whereas the “bond” between patient and therapist, as reflected in mutual trust and respect, is likely to be an essential ingredient for successful psychotherapy, it may also be the case that certain research, and perhaps clinical, agendas would be better served with a scale that focuses directly on the patient’s level of trust and respect for their clinician. For example, impairment in trust within interpersonal relationships is a central feature of borderline personality disorder (see [29] for a review). Monitoring levels of trust over the course of treatment among patients with borderline personality disorder might be a way to track improvement in this aspect of the disorder.

The goal of the current project was to propose a new scale to measure patient trust and respect for their clinicians. Our aim was to develop a scale that could be applied to psychotherapy as well as other patient-clinician contexts (e.g., medication management, case management), and be used repeatedly (e.g., at every visit) to measure changes in trust and respect over time. We report psychometric analyses of the new scale using both classical and item response theory methods, along with preliminary validity data. In regard to validity, we explored the overlap of the new scale with a measure of the alliance and the relation of the trust/respect scale to patients’ willingness to share private information with their clinician.

## Methods

### Initial development of scale

We generated items for the new scale by attending initially to the definitions of trust and respect provided by dictionaries. Because our goal was to develop a scale that might be used at every visit, brevity was essential. Items were chosen and refined during multiple group meetings of clinicians and researchers. A set of 10 items, five for trust (key words: reliable, truthful, trust, confidence in, count on) and five for respect (key words: respect, admire, have high opinion of, hold in high esteem, appreciate), were identified through these discussions for initial testing, with the potential aim of reducing to four items each for trust and respect, should the psychometric evaluation suggest that some items were less than desirable and an adequate scale could be created with the reduced number of items. Each item is rated on a 1 (*strongly disagree*) to 7 (*strongly agree*) scale, with half of the items negatively worded. The final list of items is provided in the Additional file 1 and is available for public use at no charge. After item generation, the 10-item scale was administered to patients currently in treatment at an academic-based psychiatry outpatient clinic. If patients saw more than one clinician at the clinic, they answered the questions related to the clinician they were seeing the day of the assessment.

### Patients

Participants (aged 18 or above) were recruited by research assistants in the waiting room of the outpatient psychiatry clinic. All patients were eligible regardless of the types of services they were currently receiving (i.e., medication management, psychotherapy, or both). Patients received no compensation for participation. All patients provided written informed consent and the study was approved by the University of Pennsylvania Institutional Review Board committee #8. Patient responses were anonymous and were not shared with their clinicians.

### Setting

The academic-based outpatient clinic provides psychiatric services to individuals 18 or above. Services offered include diagnostic evaluations, delivery of evidence-based psychotherapies, medication management using evidence-based decision support, and group therapies. The clinic offers specialized treatment of bipolar disorder, treatment resistant depression, anxiety disorders, substance abuse, psychosis, geriatrics, and medical-psychiatric conditions. Approximately 500 new patients each year seek services at the clinic. The clinic is staffed by 15 psychiatric residents, six attending physicians, five full-time staff psychologists, and four part-time psychologists.

### Measures

#### Alliance

A measure of the patient-therapist alliance was included to determine if the new trust/respect scale was redundant, particularly with the bond component of the alliance. The revised short-form client version of the Working Alliance Inventory (WAI-SR) [30] was used to assess the alliance. The total score of the WAI-SR has been previously reported to have an internal consistency (Cronbach’s alpha coefficient) in the range of .91 to .92 [30]. In the current study, the WAI-SR total score had an alpha coefficient of .92. Internal consistency of the 3 subscale scores were as follows: Bond: .86; Agreement on Tasks: .81; Agreement on Goals: .86. We also created a modified Bond scale, deleting the two WAI-SR items that addressed respect (“My therapist/doctor and I respect each other” and “I feel that my therapist/doctor appreciates me”) and retaining the two items that did not (“I believe my therapist/doctor likes me” and “I feel my therapist/doctor cares about me even when I do things that he/she does not approve of”). The internal consistency of this modified Bond scale was .82.

#### Willingness to share social media posts with clinician

To assess construct validity, we included an assessment of whether or not the patient was willing to share information not typically shared with a clinician as part of treatment. We hypothesized that patients would be more willing to share such information when they have higher levels of trust and respect for their clinicians. Patients were asked if they would be willing (“yes” or “no”) to share their social media posts (assuming they make such posts) with their therapist if their therapist was concerned about how they were doing.

### Statistical analyses

Initial analyses included descriptive statistics, principal components analysis to examine dimensional structure, internal consistency using Cronbach’s alpha, and corrected item-total correlations. A unidimensional Item Response Theory (IRT) method was then used to further investigate the psychometric properties of the scale using the SAS (version 9.4) IRT procedure. We implemented the Graded Response Model (GRM), which is appropriate for analyzing polytomous Likert style item responses. Within IRT, the amount of information that each item, or the test as a whole, provides is not evenly distributed across the entire continuum of the latent construct. The amount of information provided by each item is quantified in terms of the value of the slope parameter. A slope value below 1.0 was used as a threshold for detecting less discriminating items [31]. Finally, construct validity was examined by evaluating correlations between the new scale, the alliance, and patient willingness to share social media post information with their clinician. The relations of demographic variables (age, gender, minority status) to the new scale were also examined.

## Results

The set of questionnaires was completed by 218 outpatients. Women comprised 60.6% of the sample. The age range was 18 to 84, with an average age of 40.3 (*SD* = 16.0). The racial composition of the sample was 70.2% White, 23.9% Black/African American, 2.3% Native American or Alaska Native, 2.8% Asian, and 3.2% “Other or Unknown” (percentages add to more than 100% because respondents could endorse more than one race). Hispanic ethnicity was endorsed by 6.0% of the sample. Of the 218 patients, 74 (33.9%) were receiving medication management only, 32 (14.7%) were receiving psychotherapy only, and 112 (51.4%) were receiving both medication and psychotherapy services from the clinic.

The internal consistency of the total score for the initial 10 items was .91 and all items had satisfactory corrected item-total correlations, ranging from .58 to .77 (Table 1). Because brevity was a goal, we examined the internal consistency of an 8-item scale by deleting the two items with the lowest corrected item-total correlation. The internal consistency of the 8-item scale was also .91 and, therefore, we proceeded with the eight items. A principal components analysis of the eight items revealed only one eigenvalue greater than 1, with this first component explaining 74% of the overall variance. It was therefore concluded that the 8-item scale was best viewed as capturing one overall dimension. The overall mean (SD) for the total of the 8 items was 49.6 (7.9).

**Table 1:** Item Means, Standard Deviations, and Corrected Item-Total Correlations

Item	Mean	*SD*	Corrected Item-Total Correlations	
			Initial 10-item scale	Final 8-item scale
1. I respect my doctor/therapist.	6.40	1.08	.74	.72
2. I am NOT sure my doctor/therapist is reliable.	6.09	1.51	.66	.66
3. I do NOT admire my doctor/therapist.	5.95	1.55	.62	–
4. I think my doctor/therapist is truthful.	6.27	1.12	.64	.64
5. I have a high opinion of my doctor/therapist.	6.10	1.22	.77	.77
6. I do NOT have confidence in my doctor/therapist.	6.26	1.23	.76	.77
7. I do NOT hold my doctor/therapist in high esteem.	6.13	1.40	.65	.64
8. I trust my doctor/therapist.	6.13	1.21	.71	.68
9. I appreciate my doctor/therapist	6.25	1.18	.58	–
10. I do NOT feel I can count on my doctor/therapist.	6.29	1.25	.76	.77

*N* = 209. Items 2, 3 (deleted in final version), 6, 7, and 10 are reverse scored

**Fig. 1:**
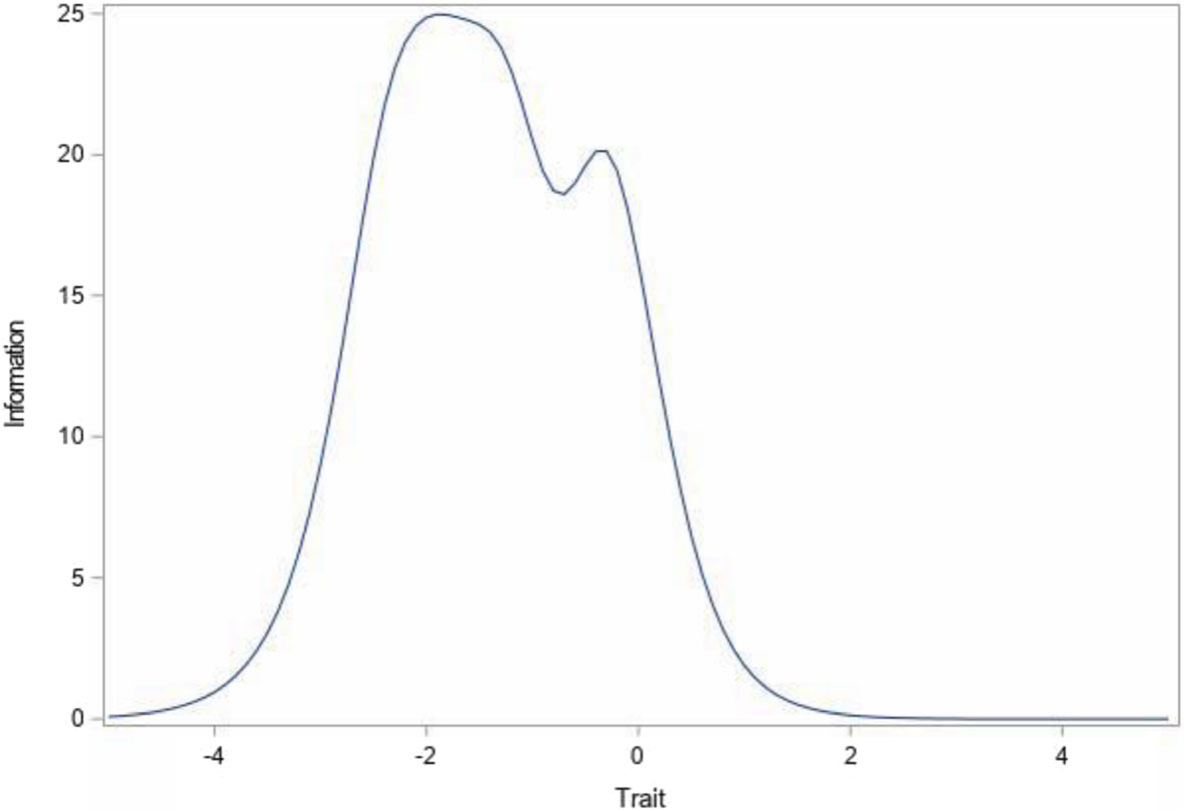
Test Information Curve for Total of 8-Item Trust/Respect Scale

Table 2 shows the slope parameters when a unidimensional GRM IRT model was fitted to the eight items. All items had slope coefficients that were considerably above the 1.0 minimum threshold rule of thumb, indicating that all items contributed information on the latent dimension and, thus, no items should be dropped. We inspected the item information curves and found that all curves were not flat at any region, which suggests that the items are reliable across the range of the latent variable. The test information curve is shown in Fig. 1. The curve peaks between − 2 and − 1 standard deviations below the mean with another small peak at the mean. Above the mean, the curve goes steeply down between the mean and about +.5 standard deviations, and at a very high level (z scores above 2.0), there is no information provided by the scale to differentiate respondents.

**Table 2:** Item Slopes from Item Response Theory Graded Response Model

Item	Slope
1. I respect my doctor/therapist.	2.48
2. I am NOT sure my doctor/therapist is reliable.	2.66
3. I think my doctor/therapist is truthful.	2.28
4. I have a high opinion of my doctor/therapist.	3.23
5. I do NOT have confidence in my doctor/therapist.	4.00
6. I do NOT hold my doctor/therapist in high esteem.	2.98
7. I trust my doctor/therapist.	2.69
8. I do NOT feel I can count on my doctor/therapist.	4.23

For assessing concurrent validity, we examined correlations of the total of the 8-item trust/respect scale with the alliance (Table 3). Although moderate in strength, correlations with the WAI-SR total score and subscale scores were well below the reliability of the 8-item trust/respect scale. In particular, the correlation with the WAI-SR Bond scale was .55, which reduced to .53 when WAI-SR items related to respect were deleted from its Bond scale. Among patients receiving psychotherapy, a small to moderate, but significant, correlation (*r* = .28; *p* = .004; *N* = 106) was apparent between trust/respect for one’s clinician and willingness to share social media posts with one’s clinician. The correlation between the WAI and willingness to share social media posts was not significant (*r* = .16, *p* = .09).

**Table 3:** Correlations of 8-Item Trust/Respect Total with Other Measures

Validity Measure	Trust/Respect Total
WAI-Bond	55**
WAI-Bond (corrected)	53**
WAI-Agreement on Tasks	43**
WAI-Agreement on Goals	51**
Willingness to Share Social Media Posts with Therapist	28*

*WAI* Working Alliance Inventory

**p < .001

*p < .005

Age (*p* = .99) and gender (*p* = .23) were not significantly associated with the total trust/respect score. However, higher trust/respect scores were apparent for White patients compared to all other patients who did not self-identify as White (*r* = .19, *p* = .009). This effect of lower trust/respect scores was particularly strong for a comparison of Black patients to all others (*r* = −.25, *p* < .001).

## Discussion

This article reports the development and validity of a trust/respect scale of patients for their clinicians. Though developed in the context of patients receiving treatment at an outpatient psychiatric clinic, the scale items were worded to have broad patient-clinician use. The final 8-item single dimension scale showed good internal consistency and reliability, and all items contributed information to the total score.

The result that a single latent dimension was mostly evident, rather than separate dimensions related to (a) trust and (b) respect, raises the question of whether the scale is primarily measuring a patient’s positive overall view of their clinician, rather than more specifically assessing their levels of trust and respect. A so-called “good guy” effect (i.e., the tendency for patients to view their therapists as generally good or bad) was hypothesized to account for the high intercorrelations among the subscales of the Counselor Rating Form by Corrigan and Schmidt [32]. To some degree, this seems likely with our new scale as well. However, the fact that the total trust/respect scale was only modestly correlated with the alliance, including the Bond scale, suggests there is a dimension partly independent of the alliance that is captured by the new trust/respect scale. In addition, the small-to-moderate association of the trust/respect scale with patient willingness to share information not normally shared with one’s clinician (social media posts), taken together with the lack of a significant association of the alliance with willingness to share social media posts, suggests that the new scale is measuring something related to trust and not simply a “good guy” effect or alliance dimension.

It is important to note that the IRT analyses revealed that more reliable information is obtained from patients that rate their trust/respect lower than the average patient. For scales with high mean scores such as the current scale (i.e., average item rating of 6.2 on a 1 to 7 scale), it is expectable that there is little reliable information in the high range. However, it is the low range of the scale that would be of more interest to clinicians and researchers. The critical question is: what is going with patients who are reporting impairments in trust/respect towards their clinician, and how can such impairments be addressed? The ability of the scale to discriminate between very positive and extremely positive trust/respect responses likely has little clinical relevance.

Scores on the trust/respect scale were not associated with age or gender. However, there was a tendency for patients who self-identify racially as White compared to other racial groups to have higher trust/respect scores. Those who racially identified themselves as Black were particularly likely to have relatively lower trust/respect scores towards their providers, a result consistent with findings of lower levels of trust towards physicians evident for non-Hispanic Blacks compared to Whites [33]. Such differences in trust can have clinical implications. For example, Black women with high blood pressure who trusted their health care providers were found to be more adherent to their prescribed antihypertensive medications than those who did not trust their health care providers [34]. With regard to mental health, lack of trust has been identified as one possible barrier for Black patients in seeking mental health services.

The new trust/respect scale opens up a number of questions that can be investigated empirically. For example: Is the combination of a positive alliance and positive patient trust/respect for their provider particularly predictive of treatment outcome? To what extent does trust/respect change over the course of treatment? What therapist actions, or clinician behaviors in other contexts besides psychotherapy, contribute to the formation and maintenance of positive trust and respect? Can alerting clinicians to negative patient reports, or ruptures, in the level of trust/respect assist such clinicians in restoring adequate trust/respect?

The current results are only preliminary and several limitations are important to note at this stage. For one, more extensive examination of the validity of the new scale is warranted. The extent to which scores are influenced by social desirability will be important to assess. Another major limitation is that we have proposed that the scale be used in a variety of patient-clinician settings, but thus far has only been investigated in the context of mental health services. A further limitation is that responsiveness to change has not been examined in the current study.

## Conclusions

With these limitations in mind, the current report provides promising preliminary data on a new, brief, trust/respect scale. The development of this scale will permit further investigation of these central, but relatively overlooked, aspects of patient-therapist and patient-doctor relationships.

## Supplementary information

**Additional file 1.** Instructions.

## Data Availability

The dataset used and analyzed curing the current study are available from the corresponding author on reasonable request.

## Abbreviations

CRF: Counselor Rating Form
GRM: Graded Response Model
IRT: Item Response Theory
WAI: Working Alliance Inventory
WAI-SR: Working Alliance Inventory – Short Revised

## References

[1] Simpson JA, Kruglanski AW, Higgins ET (2007). Foundations of interpersonal trust: handbook of basic principles. Social psychology: handbook of basic principles.

[2] Bowlby J (1969). Attachment and loss: Vol. 1 Attachment.

[3] Erikson E (1963). Childhood and society.

[4] Mikulincer M (1998). Attachment working models and the sense of trust: an exploration of interaction goals and affect regulation. J Pers Soc Psychol.

[5] Wieselquist J, Rusbult CE, Foster CA, Agnew CR (1999). Commitment, pro-relationship behavior, and trust in close relationships. J Pers Soc Psychol.

[6] Bordin ES (1979). The generalizability of the psychoanalytic concept of the working alliance. Psychotherapy (Chic).

[7] Horvath AO, Greenberg L (1989). Development and validation of the working Alliance inventory. J Couns Psychol.

[8] Webb CA, DeRubeis RJ, Amsterdam JD, Shelton RC, Hollon SD, Dimidjian S (2011). Two aspects of the therapeutic alliance: differential relations with depressive symptom change. J Consult Clin Psychol.

[9] Saunders SM, Howard KI, Orlinsky DE (1989). The therapeutic bond scales: psychometric characteristics and relationship to treatment effectiveness. Psychol Assess.

[10] Peschken W, Johnson M (1997). Therapist and client Trust in the Therapeutic Relationship. Psychother Res.

[11] Larzelere RE, Huston TL (1980). The dyadic trust scale: toward understanding interpersonal trust in close relationships. J Marriage Fam.

[12] Curtis JM (1982). The effect of therapist self-disclosure on patients’ perceptions of empathy, competence and trust in an analogue psychotherapeutic interaction. Psychother Res.

[13] LaCrosse MB, Barak A (1976). Differential perception of counselor behavior. J Couns Psychol.

[14] Barak A, Dell DM (1977). Differential perception of counselor behavior: replication and extension. J Couns Psychol.

[15] LaCrosse MB (1980). Perceived counselor social influence and counseling outcome: validity of the counselor rating form. J Couns Psychol.

[16] Ponterotto JG, Furlong MJ (1985). Evaluating counselor effectiveness: a critical review of rating scale instruments. J Couns Psychol.

[17] Tryon GS (1987). The counselor rating form – short version: a factor analysis. Meas Eval Couns Dev.

[18] Wei M, Heppner PP (2005). Counselor and client predictors of the initial working alliance: a replication and extension to Taiwanese client–counselor dyads. Couns Psychol.

[19] Khullar Dhruv (2019). Building Trust in Health Care—Why, Where, and How. JAMA.

[20] Lee TH, McGlynn EA, Safran DG (2019). A framework for increasing trust between patients and the organizations that care for them. JAMA.

[21] Blendon RJ, Benson JM, Hero JO (2014). Public trust in physicians: US medicine in international perspective. N Engl J Med.

[22] Hall MA, Zheng BY, Dugan E, Camacho F, Kidd KE, Mishra A (2002). Measuring patients' trust in their primary care providers. Med Care Res Rev.

[23] Kao AC, Green DC, Zaslavsky AM, Koplan JP, Cleary PD (1998). The relationship between method of physician payment and patient trust. JAMA.

[24] Anderson AA, Dedrick FD (1990). Development of the Trust in Physician Scale: a measure to assess interpersonal trust in patient–physician relationships. Psychol Rep.

[25] Quigley DD, Elliott MN, Farley DO, Burkhart Q, Skootsky SA, Hays RD (2014). Specialties differ in which aspects of doctor communication predict overall physician ratings. J Gen Intern Med.

[26] Rogers CR (1951). Client-centered therapy: its current practice, implications and theory.

[27] Noyce R, Simpson J (2018). The experience of forming a therapeutic relationship from the client’s perspective: a metasynthesis. Psychother Res.

[28] Lexico [Internet]. Respect; [about 1 screen]. Available from: https://www.lexico.com/en/definition/respect. Accessed 26 Feb 2019.

[29] Fertuck EA, Fischer S, Beeney J (2018). Social cognition and borderline personality disorder: splitting and trust impairment findings. Psychiatr Clin North Am.

[30] Hatcher RL, Gillaspy JA (2006). Development and validation of a revised short version of the working Alliance inventory. Psychother Res.

[31] Embretson SE, Reise SP (2000). Multivariate applications books series. Item response theory for psychologists.

[32] Corrigan JD, Schmidt LD (1983). Development and validation of revisions in the counselor rating form. J Couns Psychol.

[33] Boulware LE, Cooper LA, Ratner LE, LaVeist TA, Powe NR (2003). Race and trust in the health care system. Public Health Rep.

[34] Abel WM, Efird JT (2013). The association between trust in health care providers and medication adherence among Black women with hypertension. Front Public Health.

