# Supplementary material for: Trust and respect in the patient-clinician relationship: preliminary development of a new scale
Source: BMC Psychol. 2019 Dec 30;7:91. doi: 10.1186/s40359-019-0347-3 (PMC6937966; doi:10.1186/s40359-019-0347-3)
Supplement: Supplementary file 1 — Additional file 1. Instructions. [file 40359_2019_347_MOESM1_ESM.pdf]

Instructions

Below are a few opinions that people may have about their doctor/therapist. Circle the degree to which you agree with each given statement, just based on your most recent meeting or session.

Please respond to every question. Note that some questions are worded in terms of positive opinions and some in terms of negative opinions. Thank you for your cooperation.

Based on my last meeting with my doctor/therapist…

	Strongly Disagree	Disagree	Slightly Disagree	Neutral	Slightly Agree	Agree	Strongly Agree
**1. I respect my doctor/therapist.**	**1**	**2**	**3**	**4**	**5**	**6**	**7**
**2. I am NOT sure my doctor/therapist is reliable.**	**1**	**2**	**3**	**4**	**5**	**6**	**7**
**3. I do NOT admire my doctor/therapist.**	**1**	**2**	**3**	**4**	**5**	**6**	**7**
**4. I have a high opinion of my doctor/therapist.**	**1**	**2**	**3**	**4**	**5**	**6**	**7**
**5. I do NOT have confidence in my doctor/therapist.**	**1**	**2**	**3**	**4**	**5**	**6**	**7**
**6. I do NOT hold my doctor/therapist in high esteem.**	**1**	**2**	**3**	**4**	**5**	**6**	**7**
**7. I trust my doctor/therapist.**	**1**	**2**	**3**	**4**	**5**	**6**	**7**
**8. I feel I can count on my doctor/therapist.**	**1**	**2**	**3**	**4**	**5**	**6**	**7**
